# Biomimetic Membranes as a Technology Platform: Challenges and Opportunities

**DOI:** 10.3390/membranes8030044

**Published:** 2018-07-17

**Authors:** Claus Hélix-Nielsen

**Affiliations:** Department of Environmental Engineering, Technical University of Denmark, DK2800 Kgs. Lyngby, Denmark; clhe@env.dtu.dk; Tel.: +45-42-550-750

**Keywords:** biomimetic, aquaporin, separation, sensing, biomedicine, energy-conversion

## Abstract

Biomimetic membranes are attracting increased attention due to the huge potential of using biological functional components and processes as an inspirational basis for technology development. Indeed, this has led to several new membrane designs and applications. However, there are still a number of issues which need attention. Here, I will discuss three examples of biomimetic membrane developments within the areas of water treatment, energy conversion, and biomedicine with a focus on challenges and applicability. While the water treatment area has witnessed some progress in developing biomimetic membranes of which some are now commercially available, other areas are still far from being translated into technology. For energy conversion, there has been much focus on using bacteriorhodopsin proteins, but energy densities have so far not reached sufficient levels to be competitive with state-of-the-art photovoltaic cells. For biomedical (e.g., drug delivery) applications the research focus has been on the mechanism of action, and much less on the delivery ‘per se’. Thus, in order for these areas to move forward, we need to address some hard questions: is bacteriorhodopsin really the optimal light harvester to be used in energy conversion? And how do we ensure that biomedical nano-carriers covered with biomimetic membrane material ever reach their target cells/tissue in sufficient quantities? In addition to these area-specific questions the general issue of production cost and scalability must also be treated in order to ensure efficient translation of biomimetic membrane concepts into reality.

## 1. Introduction

The last decade has witnessed a rapid increase in new membrane materials and processes research and development. If the last century witnessed productive synergy between physics/chemistry and engineering, this century is likely to witness novel technology development driven by synergy between biology and engineering [1]. Successful advances will be based on atomistic insights gained from fundamental studies of molecular structure and function of biomolecules as well as process developments based on integrated detailed knowledge about biological tissue and organ function. 

A particularly promising area is research within membrane materials and membrane processes where new technologies are inspired directly and indirectly from the natural membrane realm [2]. Biological membranes are capable of intricate transport of water, solutes, and gasses across thin bimolecular films and can serve as an inspirational showcase for designing tailored permeability properties in large-scale polymeric matrixes or for designing nano-scale compartments for complex chemical reactions (e.g., catalysis and synthesis) [2,3]. Biological membranes are also able to perform sensing and signal transduction/energy conversion—as exemplified by retinal photoreception and photosynthesis—which may form the basis for design of biomimetic solar cells [4]. 

One manifestation in biomimetic membrane material developments is based on using bio-derived additives—either in the form of natural or engineered/modified proteins, with desired sensing and separation properties. Another manifestation is based on de novo design of membrane functionalities with design cues taken from one or more specific biological molecular structures such as ion-selective channels [5]. In both approaches the additives must be stabilized by a suitable matrix or scaffold in order to preserve the desired functionality [6].Also, process functionalities have biological correlates which may serve as inspiration: for example in the nephron, ultrafiltration occurs at the barrier between the blood and the filtrate in the glomerular capsule, and forward osmosis occurs in water reabsorption from the tubular fluids [7].

Irrespective of the membrane designs investigated, a successful translation into technology requires realistic estimations of capital and operational expenses (CAPEX & OPEX) associated with the intended application. Very often this aspect comes into consideration only after the basic science (including the selection of membrane materials and fabrication methods) behind a particular design has been developed. From an innovation point of view the worst case outcome of this sequential approach is that otherwise groundbreaking scientific results will never be translated into new technologies simply because the selected materials and production methods are not scalable and/or not cost-effective for the envisioned application. Thus, an approach where both scientific and economic aspects are considered simultaneously from very early on in a discovery-driven process may constitute a more viable path towards a commercial biomimetic product. 

## 2. Biomimetic Membrane Technology Development Status 

As of 2 July 2018, there is a total number of 3354 publications in Web of Science based on a ’biomimetic membranes’ text string search. More than 30% of these were published since 2015 with authors from USA, China, Germany, France, UK, Italy and Korea accounting for more than 85% of the publications illustrating the strong and growing global interest in the area. Many intriguing phenomena has been described inspired by the remarkable functions of naturally occurring nano-scale structures—in fields including membrane transport, catalysis, drug delivery, and electronics. Either, as their physical and chemical properties are evident a priori; or as their structures and chemistry are adjustable. However, for any nano-scale scientific result to be translated into a technology it is imperative that fabrication methods and production costs are scalable and compatible with the application intended and market segments addressed. 

Here I will briefly discuss three examples where biomimetic membrane developments have been pursued intensely over the last three decades: separation technology, energy conversion, and biomedicine. All three biomimetic areas have made great progress, but they also exemplify scientific, engineering, and commercialization challenges which must be overcome in order to translate biomimetic membrane research into technology. 

### 2.1. Membranes for Water Treatment Technology 

**Figure 1:**
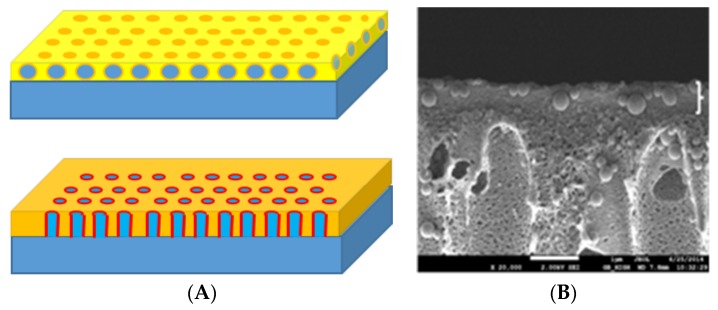
Two popular design concepts for biomimetic separation membranes [13]: (**A top**) a matrix of vesicles with reconstituted aquaporin proteins embedded in an immobilizing material (yellow) (e.g., polyamide) constitutes the selective layer formed on top of a substrate layer (blue) (e.g., polysulfone). (**A bottom**) a monolayer with the protein (red) directly embedded in a host membrane (orange) formed on top of a substrate layer (blue). (**B**) scanning electron micrograph of the design in (**A**). Scale bar 1 µm. The design in (**A top**) have also recently been used for creating membranes based on immobilized imidazole quartets [15].

A hallmark of biological membranes is their unique set of separation properties. This has led to several biomimetic attempts to design and fabricate hydrophilic membranes for separation in aqueous phases—most notably within the area of water purification. Here the use of aquaporin protein channels has attracted particular attention. The main idea behind the use of aquaporin proteins is their remarkably high water permeability (up to 10^9^ H_2_O molecules s^−1^ for certain isoforms including the mammalian AQP1 and the bacterial AqpZ [8]) reflecting the low energy barrier for transport which amounts to around 5 kcal/mol—similar to the Gibbs activation energy for water self-diffusion [9]. Thus, by incorporating aquaporin channels in a matrix impermeable to all solutes one should in principle be able to achieve a high water flux with near ideal semi-permeability. Several aquaporin membrane designs, see Figure 1, have been suggested within nanofiltration (NF) membranes [10], reverse osmosis (RO) membranes [11], and forward osmosis (FO) membranes [12], for reviews see [13,14].

The performance of any membrane design involving embedding aquaporin proteins (or any other water selective inclusion) in a host matrix will of course rely on the ability to incorporate sufficient amounts and to maintain functionality of the inclusion and the ability to create barrier properties of the matrix. Here a major issue in biomimetic membrane design based of the use of (integral) membrane proteins is the protein stability. Biological membrane protein stability is not given a priori as exemplified by the poor stability of G-protein coupled receptors in detergents [16]. Also certain proteins may require certain physical-chemical properties of the host membrane in order to function properly [17]. However, in the case of aquaporin proteins as the functional unit, proteins can be produced in high densities [18] and reconstituted in polymeric membranes with good functionality and stability [19]. 

The functionality of biological membrane channels relies on intricate nanometer-scale physical and chemical interactions and this has also led to designs for making non-biological structures with high solute/water permeability and selectivity. One early example is the design of flat, ring-shaped cyclic peptides of alternating d- and l-α-amino acids. These peptides can self-assemble into stacks and form β-sheet-like tubular structures with the amino acid side chains on the outside surface of the nanotube [5,20]—akin to the fold-structure of linear gramicidin A channels [21]. The self-assembly is driven by H-bonding between the backbone amide groups in a reversible fashion with formation and disappearance of stacked structures. Regarding channels with specific water selectivity (i.e., with concomitant rejection of ions and other solutes), π-stacked dendritic dipeptides have been investigated [22] and transport of water and protons described qualitatively [23]. Barboiu et al. have investigated imidazole compounds where H-bonded ‘quartets’ with imidazole moieties are able to form water permeable channels in lipid bilayer vesicles [24]. These channels have transport rates of ~10^6^ s^−1^ with high rejection of cations, except for protons which may be transported in the opposite direction as water (probably occurring with co-transport of chloride anions induced by vesicle swelling and proton donation from the acid dye 8-hydroxypyrene-1,3,6-trisulfonate (HPTS) entrapped inside the vesicles). Concerted water and proton transport (with exclusion of ions) has also been qualitatively demonstrated in pyridine-based (6-aminopyridine-2-carboxylic acid) oligomers stabilizing in H-bonded helical stacks [25]. 

Besides self-assembled systems also carbon nano tubes (CNTs) have been investigated as aquaporin protein channel mimics in terms of computer (Molecular Dynamics) simulations and experimental studies of water transport [26,27,28], for a comprehensive review see [29]. Potentially very high permeation rates (~10^8^ s^−1^)—exceeding Hagen-Poiseuille flow—can be obtained. However, in order to achieve high water selectivity the CNTs inner diameter has to be ~3 Angstrom, increasing the diameter to ~5 Angstrom seemingly abolishes selectivity towards ions [26]. Fornasiero et al. demonstrated that negatively charged functionalities (carboxylic groups) at the tube entrance may endow larger (sub-2-nm) CNTs with ion rejection capabilities as high as 98% for salt solutions with a Donnan z^−^/z^+^ ratio of 3 [30]. However, for KCl (where z^−^/z^+^ = 1) solutions, the maximal rejection they obtain is <55% and generally, rejection is <10% at solution concentrations >10 mM irrespective of the z^−^/z^+^ ratio [30]. Recently, experimental results on narrow CNTs with very high water permeability and selectivity (six fold higher than for the mammalian aquaporin 1 (AQP1) isoform, and no anion transport) have been reported [31]. However, the interpretation of these results have been debated illustrating the technical difficulties in quantifying single channels water permeability [32,33]. 

Common for both aquaporin proteins and artificially made water permeable structures is that high water permeability and high solute rejection is established by having a narrow hydrophilic constriction (down to ~3 Angstroms) allowing for formation of a one-dimensional water wire with H-bonding between water molecules, and bonding/polarizing interactions between water molecules and channel lumen wall, for recent reviews see [34,35]. These new types of membrane materials and components could in principle lead to fundamental improvements of membrane performance and thus may open up for new application areas both in existing and emerging market segments. However, it is not clear what the technological benefits are of radical improvements in membrane water permeability and water−solute selectivity. Do we need ultrahigh permeation or rejection? This question is of course not answerable in general terms. For desalination, which can be seen as the representative high-demanding membrane separation application, it has been argued that increased water-solute selectivity is more important than water permeability where a reasonable target for an RO desalination membrane is 2–4 L m^2^ h^−1^ bar^−1^ with near-ideal (i.e., >99.8%) rejection of NaCl at any ionic strength combined with high rejection of low molecular weight components (e.g., trace organics) [36]. For low pressure (tap water) RO membranes a target would be 9–10 L m^2^ h^−1^ bar^−1^ with NaCl rejection > 95% [37]. 

Thus, for any biomimetic water treatment membrane design to be translated into a technology there are a number of issues beyond basic performance parameters (water flux and solute rejection) which needs to be addressed. Among these is the ability to scale up production of membrane components. Another issue is the potential health hazards related to the bio/nano compounds eventually used in biomimetic membrane fabrication. Many membrane applications involve contact with food and beverage ingredients. For food contact materials, migration limits have been set for many substances based on toxicological risk assessment and these limits are now an integral part of food contact regulations. Hence migration tests should in principle capture any (unwanted) transfer of potentially hazardous compounds from food contact materials (e.g., membranes) into food. Tests typically include an overall migration limit (OML) which applies to the sum of all substances that can migrate from the food contact material to the food (or food simulant) [38]. The OML can thus be seen as a measure for the inertness of the material—and for membranes a high degree of inertness is generally desirable. In addition, Specific Migration Limits (SMLs) may have to be quantified for individual substance based on toxicological studies [38]. 

Small pore-forming molecules are known to possess antibiotic activity (e.g., gramicidin A) and it is presently not clear if any of the artificially made channel-forming molecules may also have antibiotic activity—where leakage of these compounds from the membrane is not acceptable. A similar issue may arise from the use of potentially hazardous compounds (e.g., imidazole-based pore forming compounds [24]) in substantial amounts. Finally, small nm-sized CNTs, potentially leaking from the membrane, may also present a potential health hazard being small and highly stable xenobiotic molecules [39]. 

In terms of commercialization, none of the designs involving non-biological water permeable entities have yet made any impact reflecting the abovementioned challenges in up-scaling and using these designs. Regarding aquaporin-based membranes, the Danish water technology company Aquaporin A/S is now producing and commercializing aquaporin membranes for RO and FO. The RO membrane modules are based on thin film composite (TFC) membranes in a classic spiral wound design (from small 1812 sized modules for low pressure RO applications to industrial size 4040 and 8040 modules) and the FO membrane modules are based on a hollow fiber design (with effective membrane areas up to 2.3 m^2^ per module). These membrane and modules have been tested for a number of applications. Specifically, FO membranes have been tested with respect to cleaning procedures [12,40], exposure to municipal secondary wastewater effluent [40], allowing for improved capture of carbon an phosphorous [41]. The FO aquaporin membrane concept has also been demonstrated in water recovery from molasses distillery wastewater where recovery could reach 70% compared to 35–45% for standard RO membrane treatment. Also, FO aquaporin membranes have been evaluated in a submerged osmotic bioreactor pilot experiment demonstrating the advantage of a low reverse salt flux resulting to a less severe salinity build-up in the reactor [42]. The significance of low reverse salt flux is particularly important in applications in which the concentrated FO feed solution is the desired product—such as in up-concentration of beverages/fragrances (e.g., fruit juice and coffee). This was recently demonstrated in the case of coconut milk up-concentration [43]. 

The aquaporin membrane (FO and RO) used in the applications described above is based on a design where aquaporin laden polymersomes (e.g., based on poly (2-methyl-2-oxazoline)-b-poly (dimethylsiloxane)-b-poly(2-methyl-2-oxazoline) (PMOXA-PDMS-PMOXA)) are integrated into a polyamide matrix. This design obviously raises the question if any of the constituent elements are prohibitively expensive to manufacture and/or present environmental or health risks. In terms of cost all elements in the aquaporin TFC design—except the proteins—are available in bulk commodities as they already are extensively used in classical dense membrane (e.g., RO) production. The aquaporin protein is not yet a bulk commercial product, but yields as high as 45 g/100 L fermentation has been shown with excellent purifications obtained using cost-effective detergents [43]. In terms of environmental concerns, the classical TFC membrane design is an accepted technology as exemplified by state-of-the art RO membranes. PMOXA-PDMS-PMOXA polymersomes are regarded as biocompatible [44] and aquaporin proteins are ubiquitous in edible plants such as in spinach leaves where it constitutes 20% of the total integral plasma membrane protein amount [45]. Thus, there are no apparent cost- or safety-issues related issues preventing industrial applications as such. However, there may still be commercialization challenges where the issues of stability, resistance to cleaning agents etc. are important. With respect to stability FO membrane performance has been tested and found stable over a period of at least one year [12] and this type of membrane also showed stability towards standard cleaning procedures [39]. 

Not only biological membranes have served as inspiration for designing separation membranes. Also, hydrophobic surfaces may be used in designing new membrane properties. In particular, super-hydrophobic surfaces, with a water contact angle >150° have been studied as exemplified by the lotus leaf where the hydrophobic effect arises from the surface being covered with columnar structures—papillae—about 10–20 µm in height and 10–15 µm in width coated with so-called epicuticular waxes. Based on this, several biomimetic thin membrane designs with strong hydrophobic properties have been proposed and attracted interest because of their high separation efficiency in oil/water mixtures [46]. Although such materials show potential based on laboratory scale experiments there are still major challenges to overcome. A key issue is that the extreme hydrophobicity requires that the nano-scale structures giving rise to the hydrophobicity are robust and not easily damaged by chemical and mechanical stress. Membrane fouling and physical damage will seriously impede applications. Also, it still remains to be seen how the synthesis methods can be carried out in large-scale industrial production. Methods based on controlled growth or advanced lithography may require reagent purities and production facilities which are not easily compatible with low-cost production of (expendable) membranes to be used in large-scale applications such as industrial oil/water separation. Beyond these issues there is also a more general membrane technology issue in securing effective separation of feed oil/water emulsions based on a super-hydrophobic porous material as the emulsion droplet size may span a large range: from nanometers to micrometers—and that emulsions may be highly viscous/thixotropic.

### 2.2. Membranes for Energy Conversion

In terms of large scale energy conversion conventional membranes currently play a role within the area of hydrogen separation where inorganic membranes for hydrogen separation are being investigated and used commercially. These include palladium membranes, mixed proton/electron conductive materials, and molecular sieve membranes based on silica or zeolites [47]. All these membranes are operated in the range of 300–600 °C—and in some cases at even higher temperatures at which no biological processes occur and therefore we do not have any direct biomimetic correlates. In some applications polymer membranes can be used at lower temperatures (20–80 °C) where an H_2_/CH_4_ selectivity of about 100 [48] which is of particular relevance in biogas production. 

Generally, direct biomimetic inspiration based on gas permeation in cells and tissues may hold future potentials in terms of technology developments within the area of gas separation related to CH_4_ production and CO_2_ capture—but so far developments have focused on indirect biomimetics. These developments include fabrication of mixed matrix membranes inspired by biomineralization exemplified by silica deposition in diatoms and sponges used in fabrication of CO_2_/CH_4_(N_2_) separation membranes [49], and by metal-organic frameworks where for example poly (*N*-vinylimidazole)–zinc complexes has been prepared to simulate the zinc active site of carbonic anhydrase which was subsequently immobilized in membranes for CO_2_/N_2_ separation [50]. Ye et al. have used biomimetic aquaporin FO membranes to crystallize CO_2_ by controlling the supersaturation rate for production of Na_2_CO_3_·10H_2_O. They achieved a 99.94%. purity with a water flux one to two orders of magnitude higher than other membrane approaches used for capture (e.g., membrane contactors and RO membrane crystallizers) [51]. However, until now none of these biomimetic approaches have found any commercial application. 

In terms of energy production semipermeable membrane may also find application in pressure retarded osmosis (PRO). PRO can be used to generate power from a transmembrane salinity gradient established by two different aqueous salinity streams. Originally PRO was envisaged to be applicable for sea/freshwater [52] but low energy output in this scenario has so far hampered PRO technology development which has led to consider gradients arising from hypersaline and fresh waters [53]. It is now generally accepted that in order for PRO to be economically feasible the energy density, i.e., the energy produced per unit membrane area, has to be at least 5 W/m^2^ [53]. A perquisite for reaching this density is a sufficient water flux and here aquaporin-based membranes may be advantageous given the potential for having a high water flux. FO aquaporin membranes have been tested in a model PRO study but with limited success (low water flux at salinities <2 M) [54]. Thus, more work is needed in order to clarify the potential of aquaporin membranes in PRO. Here a major issue will be to ensure low internal concentration polarization in the membrane material [55]. This can in principle be achieved by using very thin and porous membranes, but for optimal PRO energy production the membranes must be pressurized—which puts considerable demands on membrane material properties in terms of tensile strength. 

**Figure 2:**
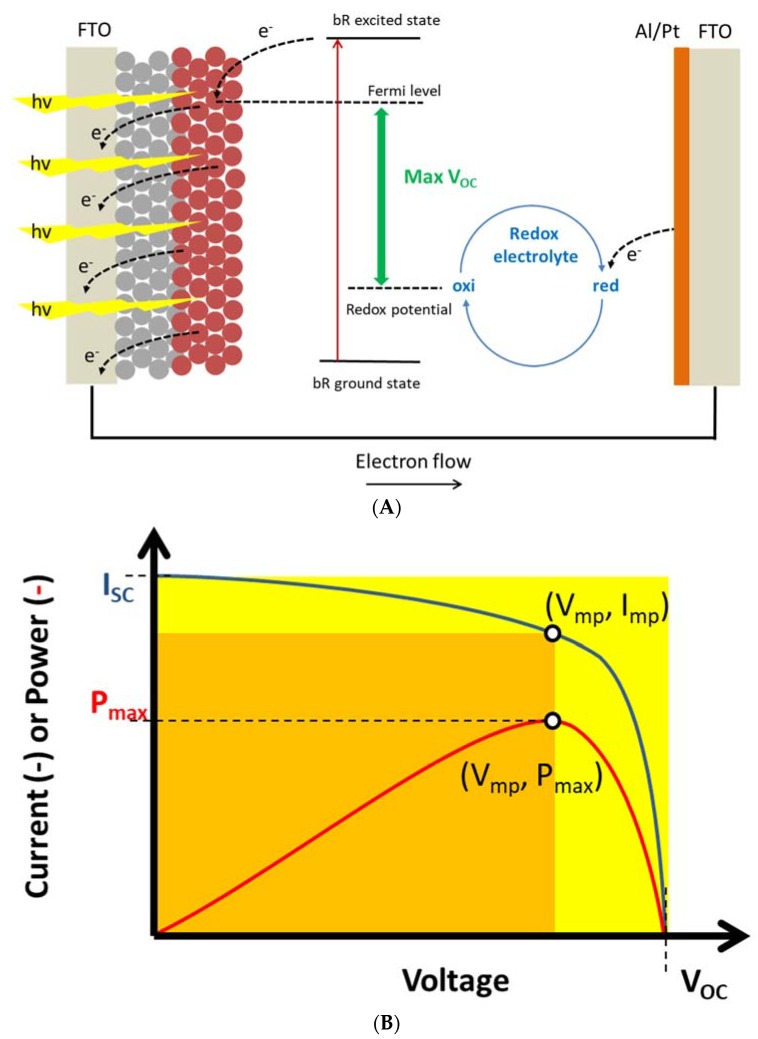
(**A**) Principal diagram of BSSC based on bR/purple membranes immobilized on TiO_2_ spheres (red) which are deposited on a fluorine-doped tin oxide (FTO) conducting glass. This acts as the working electrode with an FTO surface covered with aluminium (Al) or platinum (Pt) as counter electrode. A redox electrolyte (e.g., based on I^−^/I_2_^−^) is encapsulated between the FTO slides. The photon induced photocurrents arises from excitation of bR leading to electrons being injected into the FTO working electrode. The maximal output (V_OC_) is determined by the difference between the Fermi level and redox level. (**B**) Photo-current/photo-voltage (I/V) relation. V_OC_: open circuit voltage; I_SC_: short circuit current (at V_OC_ = 0). V_MP_: voltage at maximal power output P_MAX_. I_MP_ current at P_MAX_. The ratio between the yellow area and orange area defines the fill factor (FF), see also Table 1.

Energy conversion related to photovoltaic applications have been an area of interest in biomimetics since the first reports describing photosensitization of ZnO semiconductors using chlorophyls [56]. Strictly speaking these systems are not membrane-based but they exemplify the general idea of using a biological molecule to provide effective electron injection into a semiconductor material. This can also be achieved with the membrane protein bacteriorhodopsin (bR) where transient photocurrents generated by bR [57,58] and other light-driven pumps upon illumination have been demonstrated in systems where a surface layer (e.g., Au, SnO_2_, TiO_2_ or Indium tin oxide (ITO)) has been coated with membrane proteins (often in the form of purified protein from *Halobacterium salinarium* purple membrane) in the presence of a redox electrolyte [59,60], see Figure 2A,B.

Due to the relative ease of production and high stability of bR [61,62], both wild type and engineered bR have been investigated for optoelectronic [63] and power production (solar cell) applications [4,59]. However, no commercially available bR-based biomimetic technology exists today. In order to assess the potential of bR-based technologies one must address potential unique selling points for a bR-based technology. Provided production costs and robustness issues are solved, bR may eventually find applications within optoelectronics (e.g., in holographic storage or switching/gating devices) due to its fast differential responsivity with light-induced responses occurring within picoseconds [64]. Here the value proposition for the technology will arise from comparison with current state-of-the-art response time of semiconductor photodiodes which is in the range of 3–300 ns [65]—thus, three orders of magnitude slower. 

In terms of power production, the use of bR has been suggested as a promising concept in producing energy in bio-sensitized solar cells (BSSCs). However, the power densities obtained so far with bR have not been comparable with what has been demonstrated using chlorophyll, or organic dyes as sensitizers in conventional photovoltaic cells in which a colloidal suspension of TiO_2_ nanoparticles have been spread on conductive glass, see Figure 2A and examples in Table 1. A major challenge is to ensure a sufficient density or coating of bR onto the anodic semiconductor material. Also, the general design implies that the cathode (from which the electrons are released to interact with the redox mediator) is on top of the anode with the sandwiched electrolyte volume separating the two. This will also tend to decrease the overall efficiency. 

In some experiments bR has been compared with bacterioruberin (bRu), a linear C50-carotenoid naturally residing in biological membranes, which has photosensitizing properties—although not to the same degree as bR, see Table 1. Interestingly when both bR and bRu are present there is a cooperative effect enhancing both J_sc_, V_oc_, and η. This suggests a direct photosensitizing role of bRu having a close interaction with bR, apart from the biological role of bRu as antioxidant and regulator of membrane structural/ physical-chemical properties [66]. In other recent developments bR has been coupled to quantum dots where the quantum dots serve to absorb the energy and then transfer it to the bR via Förster energy resonance transfer in order to enhance the spectral range for the BSSC [67,68]. 

In all bR experiments above the basic mechanism (electron injection into the semiconductor) relies on changes in the protonation state of acidic and basic groups in the proteins and thus bR proton coupled electron transfer upon photoexcitation. This is therefore different as compared to the biological situation where bR function is directly related to its proton pumping capability thereby creating a transmembrane pH gradient which in turn can drive energy production in terms of ATP. Future developments using bR for energy conversion may be able to use proton gradients directly—but this will require development of biomimetic host membranes capable of maintaining these gradients once generated [75]. In any case, the fundamental—and still open—question related to bR in energy conversion remains: can we ever reach a sufficient energy density using this protein compared to dye-sensitized solar cells? A quantitative answer to this question not only relies on quantum yields of the protein but also on detailed knowledge about the specific architecture envisioned for a bR-based energy conversion: i.e., is the architecture optimal for light harvesting in terms of light access to bR and surface density of the protein. If these quantitative predictions are not successfully translated into design criteria we may never move beyond the current conceptual proof-of-principle state. 

### 2.3. Biomimetic Membranes and Membrane Processes for Biomedical Applications

Also, biological processes and cellular structures serve as biomimetic inspiration for membrane based processes. Here developments have been dominated by two complementary concepts: protocell/cell-like systems and biomimetic organelles. Both concepts may lead to and increased understanding of physiological processes, but also eventually lead to applications within medical therapy and diagnostics. 

**Figure 3:**
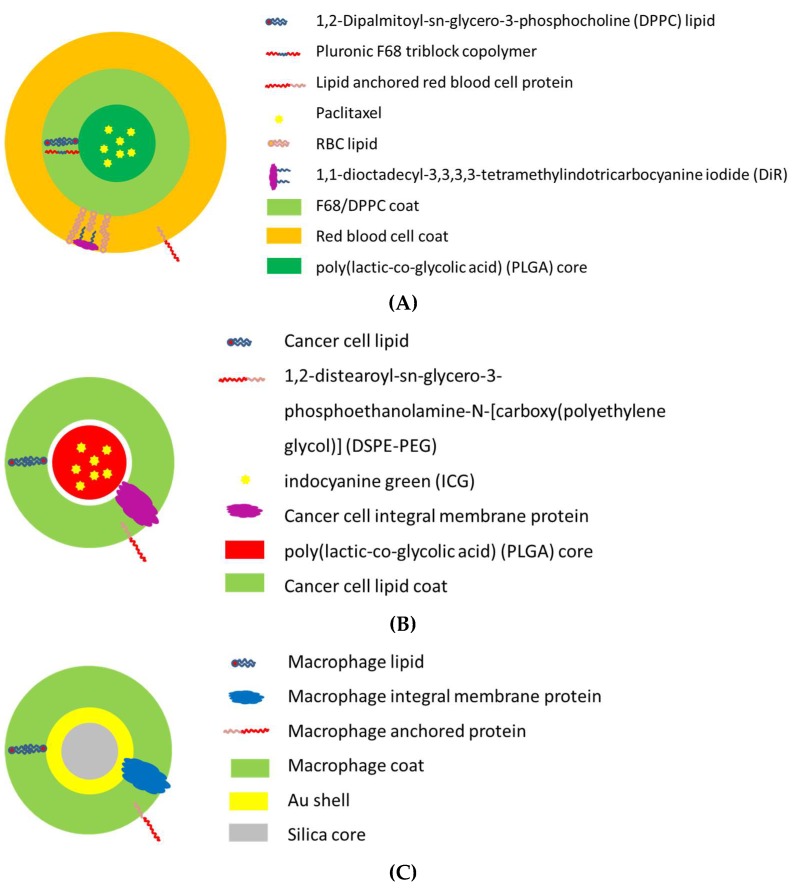
Examples of thermo-responsive nano-carrier designs with biomimetic membrane coatings where the basic mechanism of action is to induce a local temperature increase by near-infrared laser illumination to >43 °C which leads to hyperthermal tissue (tumor) destruction. (**A**) Red blood cell mimicking carrier for delivering paclitaxel (PTX) using 808 nm laser irradiation which is captured by the membrane embedded DiR dye molecule. The ensuing thermal energy triggers a DPPC phase transition and core destruction, resulting in the release of the chemotherapy medication PTX [89]. (**B**) Cancer cell mimicking carriers for delivering the diagnostic dye indocyanine dye. By utilizing binding molecules from cancer cell membranes adhesion to homologous cancer cells can be achieved while the DPPE-PEG prevents phagocytosis non-specific binding to serum proteins resulting in tumor accumulation of the nano-carrier. Irradiation at 780 nm results in energy absorption in ICG and thermal destruction of the tumor [90]. (**C**) Macrophage-camouflaged carrier where macrophage proteins provide molecular recognition with tumor proteins and the Au-coated silica core allows for energy absorption at 808 nm leading to thermal tumor destruction [91].

The area of protocell/cell-like systems for medical treatment has developed widely since the seminal discovery in 1986 that passive accumulation of nano-sized particles occurs within tumors [76]. This has spurred developments within oncology where protocell/cell-like structures with membrane mimicking surfaces has been as drug carriers based on the enhanced permeation and retention effect in solid tumors where blood vessel walls have pores with diameters up to a few hundred allowing for passage of similar sized carriers into the tumor proper [77,78]. Later the permeation concept has been modified to also include transcellular transport pathways [79]. Numerous nano-carriers or nanoparticles—many with membrane mimicking or membrane protein decorated surfaces—have been designed with the aim to conquer and destroy cancer cells and the efforts summarized in several reviews [80,81,82], see examples in Figure 3. Despite massive research efforts over three decades we still do not have a single clinical therapy realized based on nano-carriers with biomimetic properties. 

This situation has recently been described in sobering detail by Wilhelm et al. who analyzed cancer therapy nanoparticle delivery efficiency based on an extensive literature survey based on 232 independent datasets [83]. They found that only 0.7% (median) of an injected dose ever reached the tumor. Furthermore, that the choice of nanoparticle material (organic/inorganic), targeting strategy (passive/active), hydrodynamic diameter, zeta potential, shape, tumor model or cancer organ target only had modest influence on this value (i.e., the median efficiency ranged between 0.5 and 1.1%). One of the main reasons for this low efficiency is that the mononuclear phagocytotic system and the renal system will naturally tend to scavenge (and destroy) 99% of the administrated nanoparticles. This calls for even more work on cell-cell (i.e., membrane-membrane) recognition mechanisms in order to both mitigate this and to improve tumor targeting. If this fails, there is a risk that ‘biomimetic nanomedicine’ will end as a hyped research area but also a seriously underperforming technology area. 

The biomimetic organelle (multi-compartment) research has also attracted increasing attention over the last decades. Here, several strategies for obtaining these structures have been pursued including combined self-assembly and layer-by-layer assembly of amphiphilic polymers with the use of suitable (degradable) scaffolds (e.g., calcium carbonate), surface-initiated polymerization, and interfacial (emulsion) polymerization schemes resulting in formation of aqueous (liquid or gel based) droplets stabilized by surrounding polymer layers. Thus, one may create lipid-based systems (vesosomes) or mixed lipid-polymer systems (capsosomes) where a polymersome is encapsulating liposomes, for reviews see [84,85,86]. A recent example of a biomimetic compartmentalization design is a study in which artificial organelles with endogenous stimuli-triggered enzymatic activity were developed [87]. Specifically, polymersomes were used to host genetically modified outer membrane protein F porins. These proteins induce redox responsiveness with encapsulated horseradish peroxidase and activated by intracellular glutathione levels in vitro and importantly also in vivo in a zebrafish embryo model. This work is an important step towards human personalized protein therapy, but evidently more work is needed in terms of translating this proof-of-concept into medicine. Besides direct membrane biomimetics more general designs have been proposed such as a multi-membrane liquid-membrane hybrid inspired by the general structure and composition of the bacterial cell envelope. Such systems may be used in Donnan dialysis and pertraction/pervaporation systems although no technology based on these ideas have been developed yet [88].

In conclusion, even though the biomedical application area involving biomimetic membranes is indeed promising, we still need to define addressable sub-problems for which we can eventually document progress both in terms of medical/physiological effects and feasible physical–chemical mechanisms. Here, the problems of delivery and robustness must be treated in parallel with designing the mechanism of action. 

## 3. Outlook 

In terms of technological readiness of the three areas discussed here, it appears that biomimetic separation membranes are the most developed with commercial products (membranes and modules) on the market for water treatment technologies. The water market is strongly segmented which makes it a good area for identifying niches with a strong market-pull in which to introduce new technologies and obtain proof-of-market. Of particular interest are ‘high-value’ niches where the customer is willing to pay a premium price for products yet not produced in massive volumes—and in general this may be a thing to consider for commercialization of any biomimetic technology where the very first products are yet to benefit from an optimized production capability. 

However, appealing market niches may also hold challenges beyond those related to product itself. For example FO, which can be considered an emerging water technology, a successful market entry not only depends on value propositions related to the membrane performance but also on market acceptance of the FO concept itself. For other membrane application segments such as RO, successful market entry primarily depends on value propositions rooted in increased water flux/solute rejection (compared to conventional RO membranes) coupled with documented long-term stability and robustness to cleaning protocols. A special challenge is related to use of separation membranes for food and beverage applications where material safety issues must be fully addressed before relevant certifications can be obtained. 

Despite many efforts biomimetic membranes for energy conversion are still to be considered at the R&D stage. Until now, membrane protein (bR) based approaches have not been able to compete in performance with other BSSC designs in terms of photovoltaic energy efficiency. This may reflect the need for increased protein loading per unit solar cell area, but it also reflects the relatively limited spectral range for bR activation which may be augmented by including additional activation-enhancing components. These issues must be addressed and paired with realistic OPEX/CAPEX estimations before large-scale proof-of-concept studies can be realized. 

Biomimetic membranes for biomedical applications may have a huge potential, but perhaps also have the largest market entry barriers. A major barrier is the extensive in vivo validation studies needed before any of these methods will be able in the clinic. Here the focus and associated success criteria should not only be related to proof-of-concepts for specific mechanisms based on vesosomes/capsosomes or particles with membrane-mimicking coats targeting specific disease pathways and/or manifestations (e.g., tumor cells) but also on how to quantify and control accumulation/aggregation, phagocytic sequestration, and elimination via renal clearance of biomimetic membrane-based particles (and devices) in the body. Also cross-species analysis of animal models will be indispensable in translating model studies into medical applications within human care diagnostics and treatments. 

A general feature of many biomimetic mechanisms is that they may work very well at the nano-scale at which the process of interest often occur (i.e., molecular selectivity and recognition), but many large scale applications will require large sufficiently purified amounts of the functional component (e.g., membrane proteins) and/or auxiliary materials which may not be readily available. Elaborate fabrication methods may also be a hindrance for a cost-effective up-scaling of the technology. Finally, regulatory safety aspects as well as life cycle assessment must also be considered when introducing and using ‘new’ biomimetic components and processes in membrane fabrication. This is obvious for medical applications but will in fact apply to all technical applications of biomimetic technology.

## Figures and Tables

**Table 1:** **^1^** Performance data for selected BSSCs.

BSSC Substrate	Cell Area	Short Circuit Current	Open Circuit Voltage	Illumination Intensity ^j^	Efficiency	Fill Factor	Ref
	A [cm^2^]	I_SC_ [A/m^2^]	V_OC_ [V]	P [W/m^2^]	H [%]	FF -	
bR-TiO_2_^g^	0.5	0.9	0.35	400	0.002 ^a^	0.24 ^b^	[59]
bR-TiO_2_^h^	<2	2.3	0.22	600	0.03 ^c^	0.67 ^d^	[69]
bR-TiO_2_^e^	0.25	10	0.53	1000	0.35	0.66	[70]
bR-TiO_2_^e^	0.25	2.8	0.52	1000	0.09	0.62	[71]
bR-TiO_2_^f^	0.25	2.1	0.53	1000	n.a.	n.a.	[71]
bR-TiO_2_^e^	0.25	4	0.5	1000	0.11	n.a.	[72]
bRu-TiO_2_^e^	0.25	2.1	0.53	1000	0.11	n.a.	[72]
(bRu + bR)-TiO_2_^e^	0.25	4.5	0.57	1000	0.16	0.62	[72]
Cu-2-α-oxymesoisochlorin *e*4-TiO_2_^i^	1.0	90	0.52	1000	2.6	0.7 ^b^	[73]
N719 (Di-tetrabutylammonium cis-bis(isothiocyanato)bis(2,2′-bipyridyl-4,4′-dicarboxylato)ruthenium(II))-TiO_2_^e^	0.25	90.5	0.77	1000	5.9	-	[71]

^a^: calculated from Figure 11 in [59] using a linear approximation of the V_OC_-I_SC_ relationship. ^b^: FF = (V_OC_·I_SC_)_Pmax_/V_OC(max)_·I_SC(max)._ [74]. ^c^: η = V_OC(max)_·I_SC(max)_·FF/P_incoming_ [74]. ^d^: approximated by eye from Figure 11 in [69]. ^e^: with Pt-coated FTO glass as counter electrode. ^f^: with C-coated FTO glass as counter electrode. ^g^: with Al-coated FTO glass as counter electrode. ^h^: nanotube arrays. ^i^: Pt as counter electrode. ^j^: white light illumination. ^1^: Table 1 is by no means exhaustive, but serves to provide exemplary values for I_SC_, V_OC_, FF, and η in biomimetic systems allowing for direct comparison with conventional photovoltaic cells.
